# Quantification of Water Flux in Vesicular Systems

**DOI:** 10.1038/s41598-018-26946-9

**Published:** 2018-06-04

**Authors:** Christof Hannesschläger, Thomas Barta, Christine Siligan, Andreas Horner

**Affiliations:** Institute of Biophysics, Johannes Kepler University Linz, Gruberstr. 40, 4020 Linz, Austria

**Keywords:** Data processing, Imaging techniques, Biophysical methods, Permeation and transport, Biomimetics

## Abstract

Water transport across lipid membranes is fundamental to all forms of life and plays a major role in health and disease. However, not only typical water facilitators like aquaporins facilitate water flux, but also transporters, ion channels or receptors represent potent water pathways. The efforts directed towards a mechanistic understanding of water conductivity determinants in transmembrane proteins, the development of water flow inhibitors, and the creation of biomimetic membranes with incorporated membrane proteins or artificial water channels depend on reliable and accurate ways of quantifying water permeabilities P_f_. A conventional method is to subject vesicles to an osmotic gradient in a stopped-flow device: Fast recordings of scattered light intensity are converted into the time course of vesicle volume change. Even though an analytical solution accurately acquiring P_f_ from scattered light intensities exists, approximations potentially misjudging P_f_ by orders of magnitude are used. By means of computational and experimental data we point out that erroneous results such as that the single channel water permeability p_f_ depends on the osmotic gradient are direct results of such approximations. Finally, we propose an empirical solution of which calculated permeability values closely match those calculated with the analytical solution in the relevant range of parameters.

## Introduction

Alterations in the single channel water permeability (p_f_) of aquaporin’s (AQPs) are directly related to human diseases^1,2^. Alongside with AQPs, different classes of proteins like transporters^3^, ion channels^4^ and receptors^5^ represent highly efficient water pathways. To get an idea about the impact of each of these transmembrane proteins on the water permeability of the respective tissue, accurate ways of quantifying unitary water permeability values p_f_ of wild-type and mutant proteins are of particular interest and importance. Increased computer performance currently enables micro-second long MD simulations of transmembrane proteins. Despite their indisputable value to uncover molecular details of the substrate transport process, *in silico* data lack credibility without experimental validation. Furthermore, accurate p_f_ values promote further development of more precise *in silico* algorithms and models. Fresh water scarcity boosts the field of biomimetic membranes^6^ which seeks to generate highly selective and efficient lipid or polymer based filter membranes. Selectivity and permeability can be tuned by the incorporation of aquaporins (AQPs)^7–10^ and supra-^11^ or single-molecular^12^ artificial water channels. Carbon nanotubes are potential candidates as simulations already predicted high rates of waterflux^13–15^, which is only awaiting experimental verification^16^. However the performance of these biological or synthetic water channels as well as the membrane matrix itself, which is critical for channel stability and functionality^6,17^, can only be tested by accurately determining membrane water permeabilities P_f_.

Different methods^18^ exist, which are capable of retrieving information on water flux through lipid or polymer based membranes and incorporated transmembrane proteins or artificial water channels. On the one hand, *in vivo* techniques are available that can be divided into single cell swelling experiments with e.g. oocytes^19–21^ and transcellular flux through polarized cells grown on porous support^22^. While oocyte swelling experiments maybe limited by the unfolding of the oolemma^3^, measurements of transcellular flux through polarized cells offer the unique advantage of online inhibition and channel manipulation^22,23^. On the other hand, *in vitro* systems like planar lipid bilayers^24^ or lipid vesicles^25^ exist. Dilution or up-concentration of reporter ions in stagnant water layers close to planar lipid bilayers due to an osmotic water flux can be measured by ion sensitive microelectrodes^26–28^. This so called scanning electrochemical microscopy technique is time consuming and experimentally challenging, but it allows simultaneous counting of embedded electrically active channels^29–31^. However, measurements on transmembrane proteins have notably different success rates, since some proteins hardly incorporate into these planar lipid bilayers for unknown reasons. Therefore, experiments are most commonly performed with giant unilamellar vesicles (GUV)^32^ that are tens of μm in diameter or large unilamellar vesicles (LUV) with a typical size of about 100–120 nm. In contrast to GUV’s dimensions, LUV’s size cannot be determined with conventional light microscopy. A suitable signal to assess time driven size changes of liposomes is fluorescence self-quenching^33^, absorption/turbidity^34^ and scattering intensity^35^. Due to superior sensitivity and ease of use, the latter method became the standard method implemented in today’s stopped-flow experiments. The scattering intensity depends on the vesicle’s size and the refractive index. After a hyperosmotic shock, the concentration of entrapped osmolytes increases due to a decrease in vesicle volume. This increase in inner osmolarity increases the vesicle’s refractive index which elevates their light scattering ability. At the same time, the loss of vesicle size, reducing its scattering ability, counteracts this effect. To relate vesicle volume to scattering intensity, empirical approximations ranged from double logarithmic^25^, over quadratic^36^ to simple linear^37^ relations. We found that the computed solution of the scattering intensity in dependence of vesicle volume is well described by a second degree polynomial function of the vesicular volume^38^.

In a stopped-flow device, vesicles in osmotic equilibrium are subjected to a hyperosmotic buffer with impermeable solutes of concentration *c*_*out*_ at time zero. The vesicle shrinkage depends on the water permeability of the vesicular membrane P_f_:

1 $$\frac{dV(t)}{dt}=A{P}_{f}{V}_{w}({c}_{in}(t)-{c}_{out})$$

2 $${c}_{in}(t)=\frac{{V}_{0}}{V(t)}{c}_{in,0}$$

where V_w_, V_0_, A, c_in,0_ and c_out_ are the molar volume of water, vesicle volume at time zero, constant surface area of the vesicle, the initial osmolyte concentration inside the vesicles, and osmolyte concentration in the external solution, respectively. We assume that the osmolarities change indirectly proportional to the vesicle volume. This is justified by an almost linear relation between concentrations and osmolarities for various substances in the relevant concentration range^39^.

**Figure 1:**
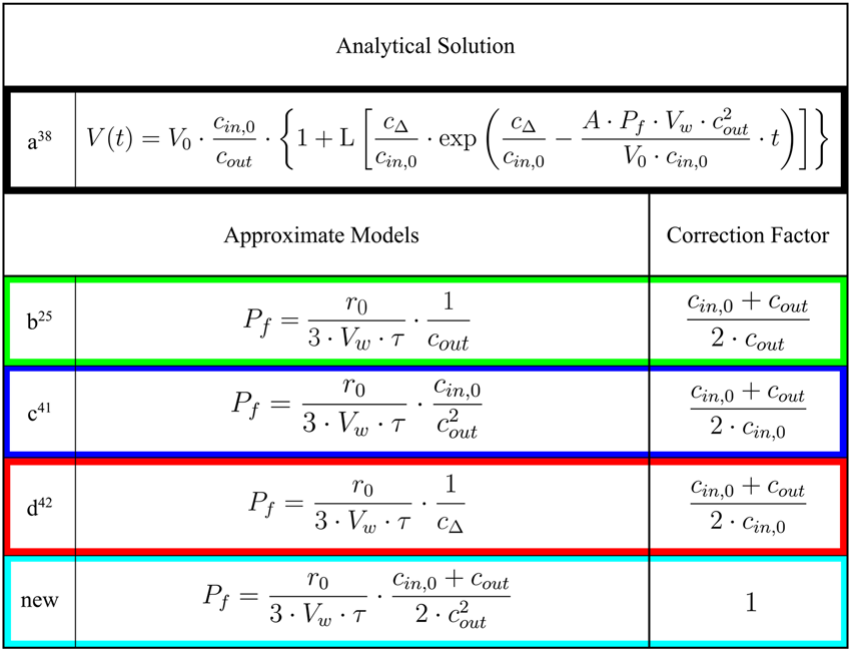
Models used to estimate P_f_ values from light-scattering data. Water permeability’s P_f_ of exponential approximations depend on the outer and initial inner osmolarities. Correction factors can be used to recalculate erroneous P_f_ values. The different models are color coded throughout the entire manuscript.

**Figure 2:**
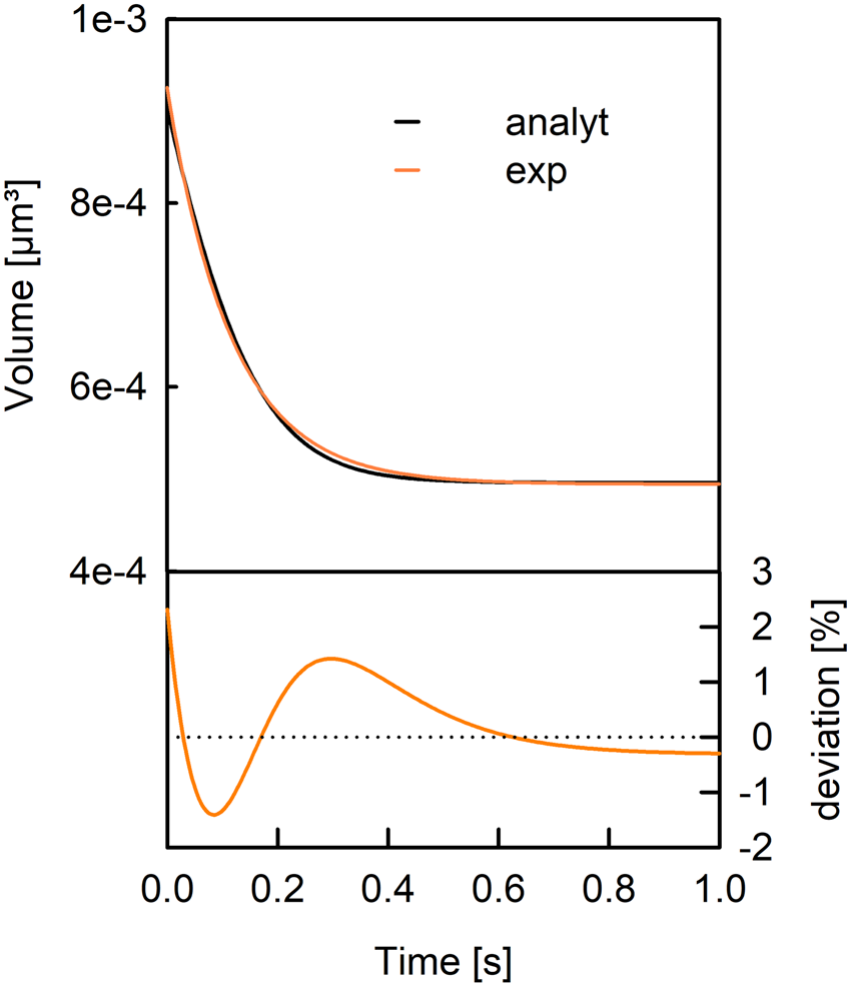
Comparison of an exponential fit to the analytically calculated decrease in vesicle volume. The fit of an exponential function with initial and final volume as fit parameter (orange line) to the analytical solution (black) of a vesicle with a radius r_0_ of 60 nm exposed to a hyperosmotic buffer at time zero. The deviation of the exponential fit to the analytical solution is depicted below. An initial inner osmolarity of 200 mOsm, an outer osmolarity of 300 mOsm and a permeability of 20 µm/s are used.

Several decades ago these differential-algebraic equations were solved numerically^36,40^ to calculate V(t) for various P_f_ values. Time constants τ of mono-exponential fits to the permeability dependent V(t) were matched to experimentally fitted τ values to obtain P_f_. However, in the meantime different approximations^25,41,42^ were proposed to calculate P_f_ directly from τ. These models only differ in the way the osmotic conditions in the vesicles interior and exterior are considered (Fig. 1b–d). An analytical solution exists for Eq. (1) at hyperosmotic conditions^38^ (Fig. 1a), but still approximations are applied. Even though an exponential function (Fig. 2, black line) is not an exact solution to Eq. (1), it resembles the analytical solution. However, these approximations based on exponential fits to the scattering data lead to systematic errors and false positive effects as described below. Therefore we question all published approximations of estimating P_f_ from light scattering traces of vesicle shrinkage and search for a new relation based on the time constant of exponential fits to the scattering data. To do so, we compare the discrepancies between the different methods by combined experimental and computational approaches. Permeability values obtained from different approximations are analyzed in respect to the absolute concentration of osmolyte, the osmotic gradient, the actual permeability and the vesicle radius. Finally we propose a new relation based on τ values which closely resembles P_f,analyt_ calculated with our analytical solution.

## Results

**Figure 3:**
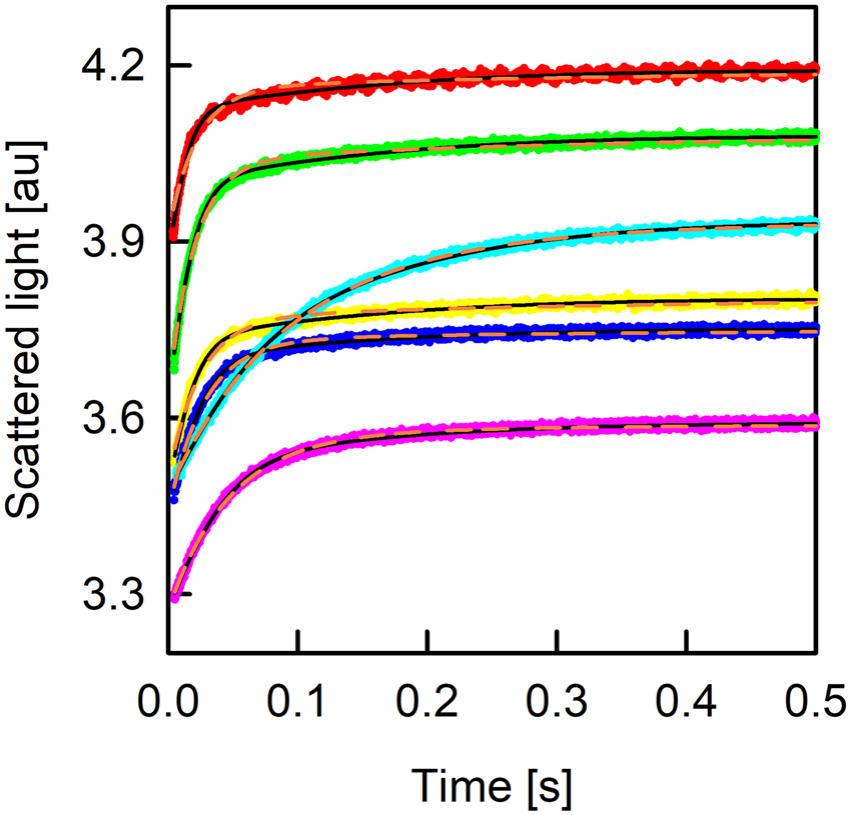
The osmotic shrinkage of GlpF containing proteoliposomes. Stopped-flow raw data (colored spline lines), fits (black lines) according to Eqs (7) and (8) and exponential fits (orange short dashed) for varying amounts of GlpF (on average number of monomers/PL; 4.5 - red line; 4.1 - green line; 3.5 - yellow line; 2.7 - blue line; 2.0 - pink line; 1.5 - cyan line). Equal volumes of vesicle suspension (100 mM NaCl, 20 mM MOPS, pH 7.5) and hyperosmotic solution (300 mM sucrose, 100 mM NaCl, 20 mM MOPS, pH 7.5) were mixed at 4 °C.

**Figure 4:**
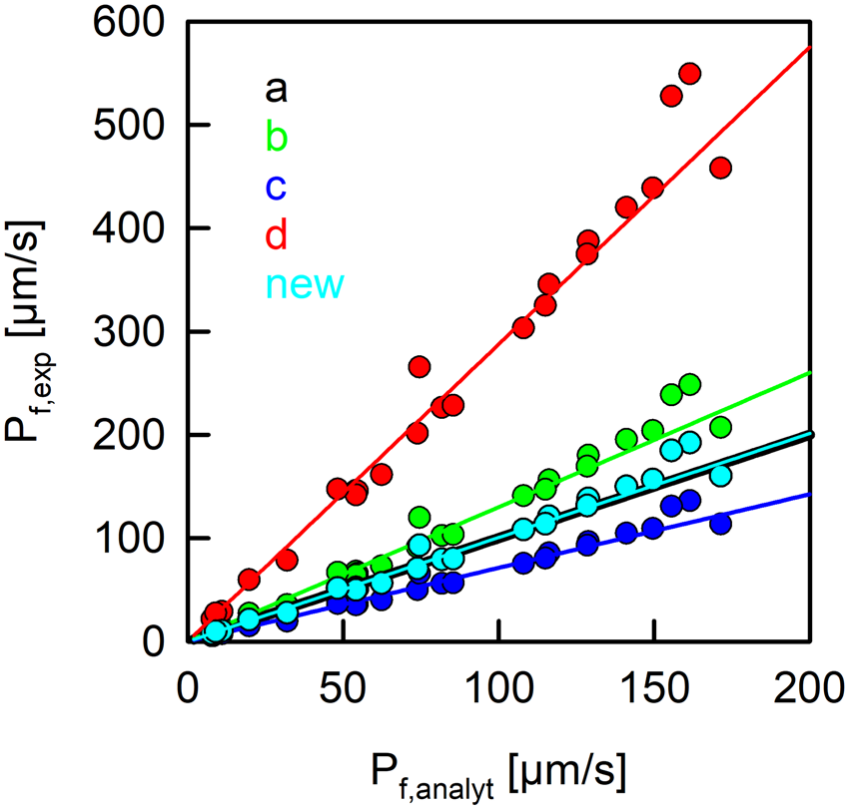
Correlation of permeabilities obtained from exponential fits, P_f,exp_, and the analytical solution, P_f,analyt_. Permeability values obtained by the fit of an exponential function to the analytical solution as in Fig. (2) (solid lines) deviate depending on the model used (b: Π = c_out_^−1^, green; c: Π = (c_out_ − c_in,0_)^−1^, red; d: Π = c_in,0_·c_out_^−2^, blue). Our new relation (new: Π = (c_out_ + c_in,0_)·(2·c_out_^2^)^−1^, cyan) closely tracks the black line for the analytical solution. Dots represent permeability values obtained by fitting scattering raw data obtained from GlpF containing proteoliposomes (as in Supplementary Fig. S1) either with an exponential fit together with the different approximations (y-data) or with the analytical solution (x-data). The buffer conditions were the same as in Fig. (3).

The rate of vesicle shrinkage depends on P_f_ (Eq. 1). This differential equation was analytically solved by Horner *et al*.^38^ (Eq. 7) and is depicted in Fig. (2) (black line). It may be fitted by an exponential function (Fig. 2, orange line) even though the latter is not an exact solution to Eq. (1). Three different approximations (Fig. 1b–d) connect P_f_ with the exponential time constant τ (Eq. 6) of vesicle shrinkage. They only differ in the way c_in,0_ and c_out_ are considered. To test these models for their deviation from the analytical solution depending on P_f,analyt_, we first overexpressed, purified and reconstituted the aqua-glyceroporin of *E.Coli*, GlpF, into large unilamellar vesicles (LUVs). We subjected these proteoliposomes to a hyperosmotic solution and followed the decrease in vesicle volume by light scattering (Fig. 3). The raw data are either fitted by the analytical solution (Eqs 7 and 8) considering two vesicle populations^38^ or by a double exponential function where the slow time constant corresponded to bare lipid vesicles and the fast time constant to GlpF containing vesicles. By adjusting the protein to lipid ratio during reconstitution, we experimentally varied the water permeability calculated with the analytical solution, P_f,analyt_, between 5 and 175 µm/s. The deviation of P_f,exp_ from P_f,analyt_ depends crucially on the approximation used (Fig. 4). However, the relative deviation is independent of P_f,analyt_. Comparing these approximations (Fig. 1b–d) for the relation between τ and P_f,exp_ revealed that the arithmetic mean of the two approximations (b, c) closely resembles P_f,analyt_ in the investigated range of parameters with a maximal error of 5% (Fig. 1, new):

3 $${P}_{f}=\frac{{r}_{0}}{3\cdot {V}_{w}\cdot \tau }\cdot \frac{{c}_{in,0}+{c}_{out}}{2\cdot {c}_{out}^{2}}$$

To computationally verify this result we computed the analytical solution V(t) for a series of P_f_ values between 2 and 200 µm/s. The time constants of the exponential fits to the data and the three published approximations (Fig. 1b–d) as well as our newly found approximation (Fig. 1, new) were used to calculate the corresponding membrane permeabilities P_f_. These permeability values are consistent with the experimentally found discrepancy between the approximations based on exponential time constants and the analytical solution (Fig. 4).

**Figure 5:**
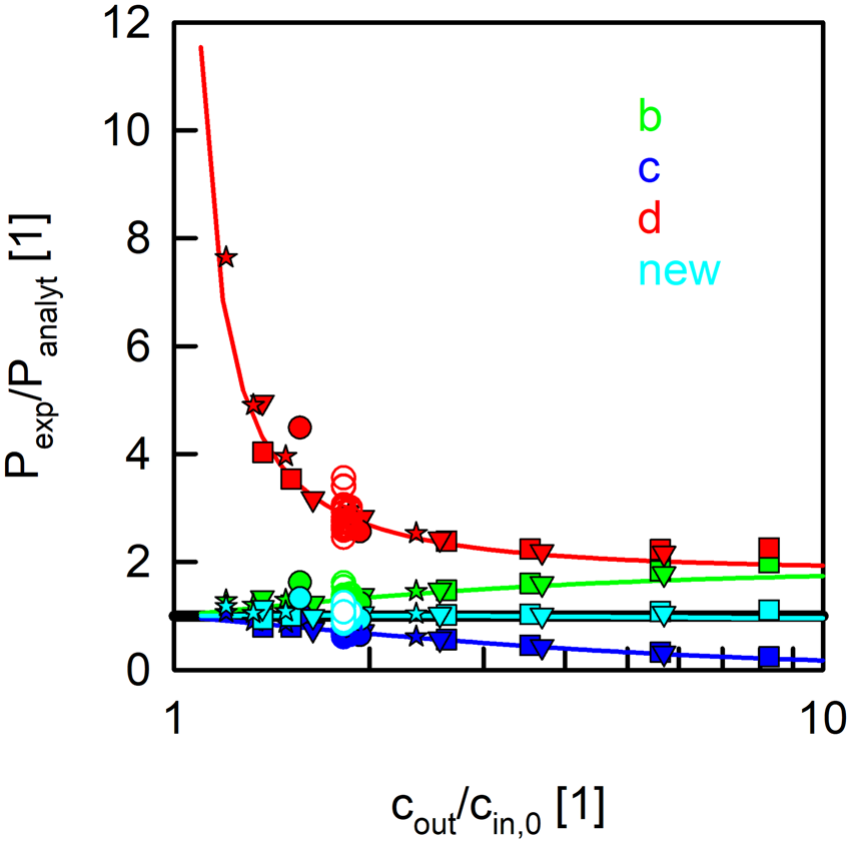
Permeabilities calculated from the time constant of exponential fits, P_f,exp_, depend on the gradient factor G = c_out_/c_in,0_. Permeability values are normalized to P_f,analyt_. The analytical solution (black line) is independent of G. Computed permeability values obtained by a fit of an exponential function to the analytical solution as in Fig. (2) (solid lines) deviate from P_f,analyt_ depending on the model (color code as in Figs 1 and 3) used. Control vesicles with four different c_in,0_ (filled square, 97 mOsm; filled triangle, 171 mOsm; filled star, 538 mOsm, filled circles, 919 mOsm) are subjected to different c_out_. Proteoliposomes containing GlpF from Figs (3) and (4) (empty circles) comprising a c_in,0_ of 200 mOsm are subjected to a hyperosmotic solution containing 150 mM sucrose (365 mOsm). Computational data were generated with control vesicles by keeping c_in,0_ constant at 200 mOsm and varying c_out_ between 220 and 2000 mOsm.

Next, we studied the deviation of P_f,exp_ for different inner and outer osmolarities. Therefore, we varied c_in,0_ (97 to 919 mOsm) and c_out_ (133 to 1778mOsm) of control vesicles (bare lipid vesicles) resulting in a gradient factor G = c_out_/c_in,0_ between 1.2 and 8.3. These experiments (Supplementary Fig. S1) revealed that the deviation of the approximate models from the analytical solution do not depend on the absolute inner osmolarity c_in,0_, but only on the gradient factor G (Fig. 5). Computational analysis confirmed these results (Fig. 5 and Supplementary Fig. S2). The deviation of permeabilies P_f,exp_ obtained from exponential fits to the analytical solution P_f,analyt_ for the osmotic shrinkage of vesicles with radius r_0_ = 60 nm and a water permeability of 20 µm/s depends on the relative inner and outer osmolarities but not their absolute values (Fig. 5 and Supplementary Fig. S2). To ensure comparability with experimental data we normalized all permeabilities P_f,exp_ with P_f,analyt_. By calculating G for the proteoliposomes shown in Figs (3) and (4) and plotting them into Fig. (5), we strengthen the argument that the relative deviations between P_f,exp_ and P_f,analyt_ are independent of P_f,analyt_. The normalized permeability values perfectly match our control measurements. Hence Fig. (5) illustrates that the accuracy of the different models (Fig. 1b–d) only depends on G and in the range of investigated ratios, P_f,exp_ may be decreased or increased compared to P_f,analyt_ by a factor of six or several orders of magnitude, respectively. Our new approximation (Eq. 3, Fig. 1, new) gave similar values to P_f,analyt_, independent of the experimental conditions. The simple dependence of the error on the osmotic conditions allows recalculating an erroneous P_f_ obtained by one of the old models (Fig. 1b–d) with the corresponding correction factor depicted in Fig. (1).

## Discussion

Direct p_f_ measurements are subject to large inherent technical difficulties. They include effects of stagnant water layers in membrane vicinity and uncertainties in the actual channel density^43^. Additional errors are introduced by using approximations (Fig. 1b–d) to calculate P_f_ from the time constant τ of a single exponential fit to the scattering data. For example model d depicted in Fig. (1) produces an error that depends on the osmotic gradient^44,45^ (see Fig. 5d), which can neither be explained by a structural resistance of LUVs to volume changes^33^, nor by an intra-vesicular unstirred layer^35,38^. Alternative methods, like scanning electrochemical microscopy^29^ and fluorescence self-quenching experiments combined with a numerical solution^33^, confirm that P_f_ is independent of the osmotic gradient. Still this model has widely been used to report for example water permeabilities for KcsA^4^, several AQPs^8,17,42,46–52^ and artificial water channels^11,12^. Models b and c shown in Fig. (1) exhibit a smaller relative error when changing the ratio c_out_ to c_in,0_. Figure (5) depicts that approximation b and c represent a systematic over- or underestimation of P_f,analyt_ with an increasing relative osmotic gradient. To conclude, varying G from 1 to 10, models b, c and d produce a systematic relative error up to a factor of 2, 6 and several orders of magnitude, respectively. We further demonstrate by means of computational and experimental data that the deviations of P_f,exp_ from P_f,analyt_ do not depend on P_f,analyt_ or c_in,0_ but solely on G, the ratio c_out_/c_in,0_. This is evident as models b to d only differ in the way they consider c_out_ and c_in,0_.

The above discussed approximations tend to systematically deviate from P_f,analyt_, due to oversimplifications in the respective derivations. For example, model b linearly approximates V(t) based on the initial change in volume^25^. The elaborate calculation yields a similar result as a heuristic approach that assumes an exponential volume change with the final volume depending on the ratio c_in,0_/c_out_:

4 $$V(t)={V}_{0}\cdot ((1-\frac{{c}_{in,0}}{{c}_{out}})\cdot \exp (-\frac{t}{\tau })+\frac{{c}_{in,0}}{{c}_{out}})$$

Comparing the derivative of Eq. (4) at time zero

5 $${\frac{dV(t)}{dt}|}_{t=0}=-\frac{{V}_{0}}{\tau }\cdot (1-\frac{{c}_{in,0}}{{c}_{out}})$$

with the initial change in volume (Eq. 1 at t = 0), one obtains approximation b. Yet the best-fit global time constant does not necessarily give the best fit to the initial rate of volume change which ill-poses the assumption of a strict exponential shrinkage. But, the flatter the kinetics, the better this approximation is. An example is provided by experiments with small relative osmotic gradients (Fig. 5). Approximation d can also be obtained heuristically, but with the assumption that the volume drops exponentially to zero instead to the ratio c_in,0_/c_out_. Especially at very low osmotic gradients or gradient factors close to 1 model d leads to a divergent solution since only for large gradients c_in,0_/c_out_ approaches zero. Approximation c results in rather reliable values for small relative osmotic gradients as its derivation relies on small volume changes^41^ (Fig. 5).

Generally P_f_ is independent of the initial vesicle radius r_0_^38^. Any vesicle population with a unimodal radius distribution can be fitted by considering only one mean vesicle diameter. Still under- or overestimation of r_0_ leads to an error in P_f_, which linearly depends on the ratio of assumed to real vesicle radius (Supplementary Fig. S3). Hence, r_0_ must be known with even higher accuracy to resolve small changes in P_f_. It is important to note that for a scattering technique it is mandatory to estimate the mean radius of the intensity distribution and not the mean radius of the volume or particle distribution.

Finally, we established a new relation to calculate accurate P_f_ values from the time constant of an exponential fit to scattered light of vesicle shrinkage. Our new model (Fig. 1, new) yields an osmotic gradient independent permeability value consistent with the analytical solution of Eq. (1), which was already published in 2015. However, the analytical solution was only applied in our work group so far to calculate p_f_ values of AQP1, GlpF, AqpZ, KcsA^38^, AQP4^53^ and hSGLT1^3^. We attribute this to the fact that the Lambert function as part of the analytical solution is not implemented in common tools for stopped-flow data analysis. This survey enables the community to obtain correct permeability values from exponential fits or recalculate previous erroneous results.

## Methods

### Protein Overexpression, Purification and Reconstitution

The sequence of GlpF (*Escherichia coli*) is inserted in a pTrc plasmid, which is transformed into C43 (DE3) cells. The cells are grown in LB overnight, diluted 40-fold and grown until OD 0.6. Expression is induced by 1 mM IPTG for 3 hours. Cells are harvested and pellets frozen at −80 °C. Purification and Atto488 labeling is performed as we described elsewhere^38,54,55^. In brief, cell pellets are lysed and the pelleted cell fraction is solubilized in a detergent buffer. After the removal of the insoluble material by ultracentrifugation, the supernatant is further purified using affinity (Ni^2+^-column). GlpF is reconstituted into proteoliposomes (PLs) with small modifications as previously described^38,54,55^. *E.coli* polar lipids (PLE, Avanti Polar Lipids) doped with 0.004 m% Atto633PPE are dried on a rotary evaporator. The thin lipid film is rehydrated in Reko buffer (100 mM NaCl, 20 mM MOPS, 1.4% OG, pH 7.4) to attain a final lipid concentration of 20 mg/ml. Subsequent to bath sonication, the clear suspension is incubated with equal amounts of protein diluted in Reko buffer at room temperature for an hour. With stepwise addition of Biobeads SM-2 (Bio-Rad), we remove the detergent within 36 hours. PLs are harvested by ultracentrifugation. The resuspended vesicles are centrifuged to remove aggregates and put through 21 extrusion cycles stacked with two polycarbonate filters with 100-nm pore sizes using a mini-extruder from Avanti Polar Lipids. This results in a unimodal radius distribution as seen from dynamic light scattering measurements. In addition protein reconstitution results in a fraction of bare lipid vesicles and a fraction of proteoliposomes with a heterogeneous but very sharp distribution of proteins between the lipid vesicles^38^. Control vesicles are treated similarly. All samples are assayed without delay.

### Bare Lipid Vesicle Preparation

Large unilamellar vesicles (LUVs) were prepared from an *E.coli* polar lipid extract (PLE) mixture in chloroform as described elsewhere^56^. In brief, PLE was dried on a rotary evaporator, hydrated in a solution containing 100 mM NaCl and 20 mM MOPS buffered at pH 7.4, and extruded through 100 nm polycarbonate filters as described above. The final stock solution subsequently contained 10 mg/ml lipids. For experiments with different c_in,0_ the NaCl concentration was varied accordingly.

### Stopped-Flow & Data Analysis

PLs and LUVs are subjected to a hyperosmotic solution in a stopped-flow apparatus (SFM-300, Bio-Logic, Claix, France) at 4 °C. As previously described^3,4,38,53^, we monitor the intensity of scattered light at 90° at a wavelength of 546 nm. To calculate water permeability values from light scattering we use our recently found analytical solution^38^ and three common approximations^25,41,42^ as well as our new approximation all based on single-exponential functions with a time constant τ

6 $${P}_{f}=\frac{{r}_{0}}{3\cdot {V}_{w}\cdot \tau }\cdot {\rm{\Pi }}$$

where depending on different models Π is equal to c_out_^−1^, (c_out_ – c_in,0_)^−1^, c_in,0_·c_out_^−2^ or (c_in,0_ + c_out_)/(2· c_out_^2^). The analytical solution for Eq. (1) at hyperosmotic conditions can be written as^38^:

7 $$V(t)={V}_{0}\frac{{c}_{in,0}}{{c}_{out}}\{1+L(\frac{{c}_{{\rm{\Delta }}}}{{c}_{in,0}}exp(\frac{{c}_{{\rm{\Delta }}}}{{c}_{in,0}}-\frac{A{P}_{f}{V}_{w}{{c}_{out}}^{2}}{{V}_{0}{c}_{in,0}}t))\}$$

where c_Δ_, and L are the incremental osmolyte concentration in the external solution due to osmolyte addition (cΔ = c_out_ − c_in,0_) and the Lambert function defined by L(x)·e^L(x)^ = x, respectively. This solution only holds for hyperosmotic conditions since the Lambert function is not defined for hypoosmotic solutions where *c*_*out*_ < 0.841 · *c*_*in*,0_. Anyway, the surface area of a vesicle can only expand by a few percent^57^ before the vesicle bursts and the condition of a constant amount of encapsulated osmolytes is violated. The vesicle volume is experimentally accessible by measuring the scattering intensity. Careful treatment of scattering intensity I(t) of vesicle shrinkage by employing the Rayleigh-Gans-Debye relation and acknowledging the change in size and refractive index revealed that a second order Taylor series with coefficients a, b, d is of high accuracy (see SI of Horner *et al*.)^38^:

8 $$I(t)=a+b\cdot V(t)+d\cdot {V}^{2}(t)$$

However, in the relevant range of r_0_ (~30 to ~100 nm) Eq. (6) can be approximated with a linear dependence of V(t) on I(t) with acceptable accuracy (Fig. S1^38^). In combination with our new approximation (Eq. 3, Fig. 1, new) this results in P_f_ values deviating only a few percent from P_f,analyt_ (Fig. 4, cyan dots). In addition we take into account the fraction of bare lipid vesicles in a proteoliposome preparation as described elsewhere^38,53^. Osmolarities of c_in,0_ and c_out_ were routinely checked using a vapor pressure osmometer 5500 from (Wescor, EliTechGroup, Utah).

### Computational Analysis

All mathematical computation is done with Mathematica 11.2 (Wolfram Research 2017). A function was implemented which takes c_in,o_, c_out_, P_f_ and r_0_ as an argument, tabulates the numerical V(t) values of the analytical solution (Eq. 7) to these arguments and returns the time constant τ of an exponential fit to the numerical values. An exponential fit to the analytical solution with initial and final volume as fit parameters violates the initial volume change (Eq. 1) as the fit overestimates V(t) at time zero as seen from the initial slopes in Fig. (2) However, we are only interested in the kinetic parameter τ, which is subsequently used in conjunction with Eq. (6) to derive approximate P_f,exp_ values. These purely computationally derived P_f,exp_ values are subsequently compared to P_f,exp_ values obtained by the time constants of exponential fits to the scattering raw data, which comprises a linear dependence of V(t) on I(t). If not varied for systematic comparison, P_f,analyt_ and r_0_ are set to 20 µm/s and 60 nm. If not stated differently c_in,0_ is set to 200 mOsm and c_out_ to 300 mOsm.

### Availability of materials and data

The datasets generated during and/or analyzed during the current study are available from the corresponding author on reasonable request.

## Electronic supplementary material

Supplementary Information
