# Author Correction: Quantification of Water Flux in Vesicular Systems

**DOI:** 10.1038/s41598-020-57825-x

**Published:** 2020-01-15

**Authors:** Christof Hannesschläger, Thomas Barta, Christine Siligan, Andreas Horner

**Affiliations:** Institute of Biophysics, Johannes Kepler University Linz, Gruberstr. 40, 4020 Linz, Austria

Correction to: *Scientific Reports* 10.1038/s41598-018-26946-9, published online 04 June 2018

This Article contains an error in Figure 1, where the correction factor for approximate model d is incorrect. The correct Figure 1 appears below.

**Figure 1:**
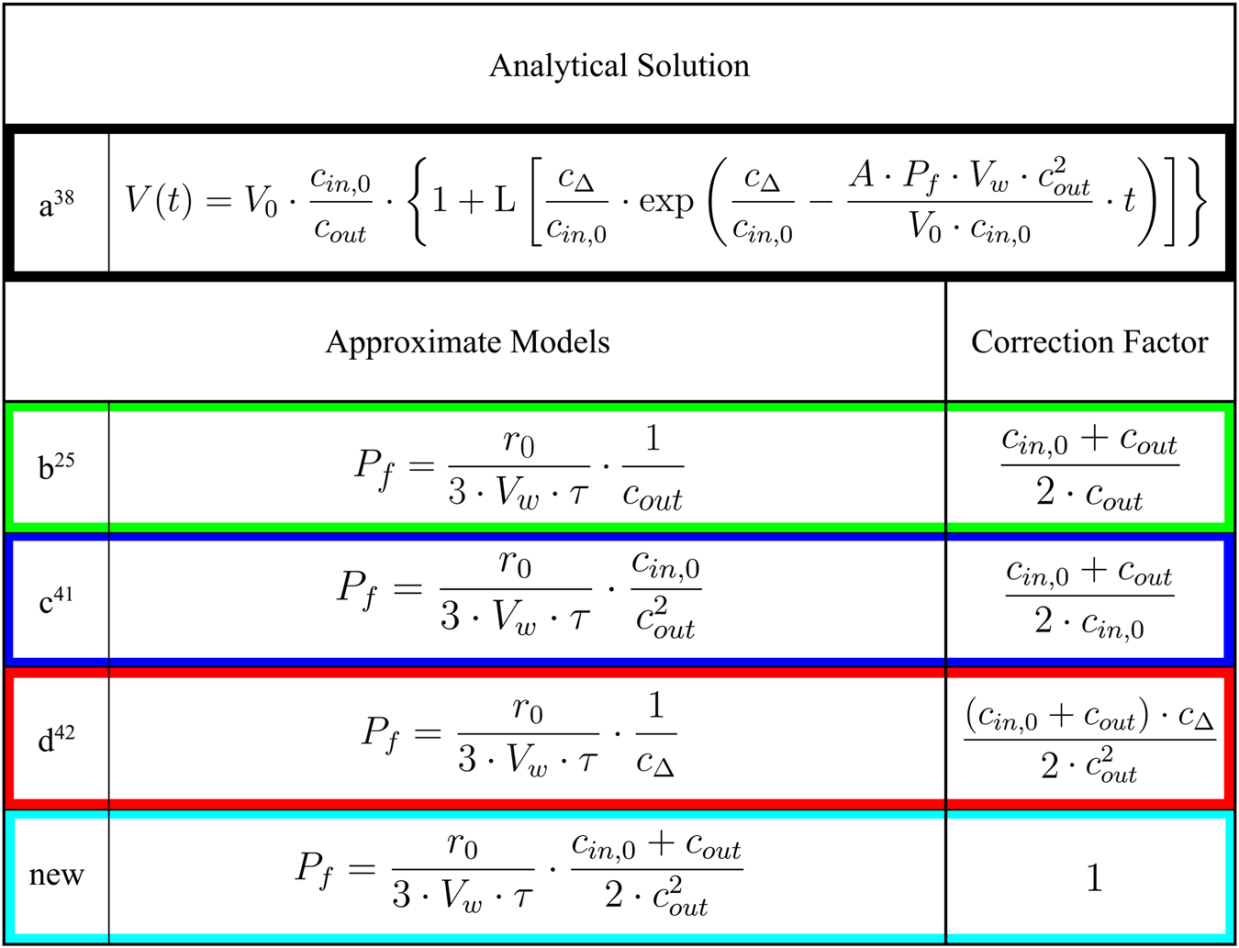
.

