# Supplementary material for: Quantification of Water Flux in Vesicular Systems
Source: Sci Rep. 2018 Jun 4;8:8516. doi: 10.1038/s41598-018-26946-9 (PMC5986868; doi:10.1038/s41598-018-26946-9)
Supplement: Supplementary file 1 — Supplementary Information [file 41598_2018_26946_MOESM1_ESM.docx]

**Supplementary Information**

**Quantification of Water Flux in Vesicular Systems**

Christof Hannesschläger, Thomas Barta, Christine Siligan, Andreas Horner^*^

From the Institute of Biophysics, Johannes Kepler University Linz, Gruberstr. 40, 4020 Linz, Austria;
^*^Correspondence should be sent to andreas.horner@jku.at


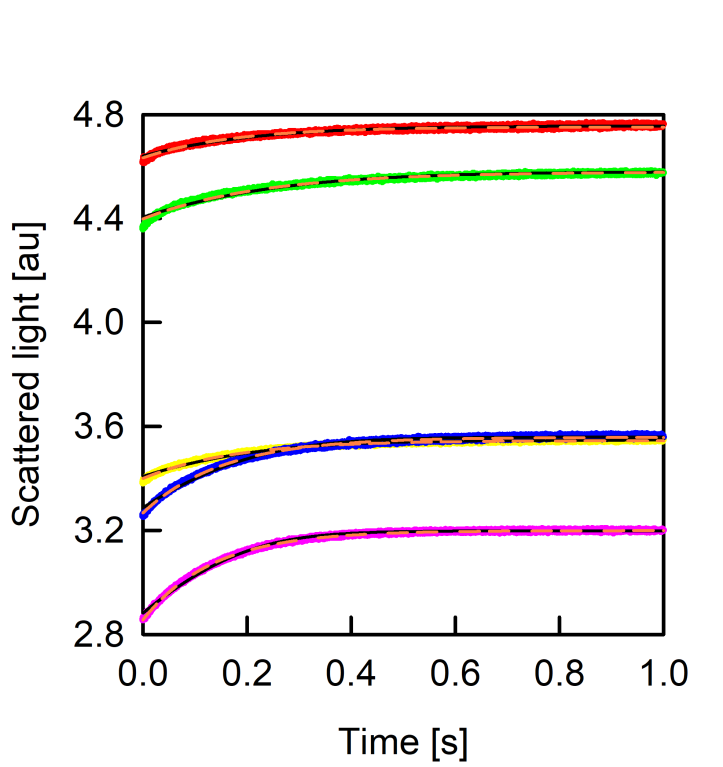


**Supplementary Figure S1. The osmotic shrinkage of LUV’s.** Exemplary stopped-flow raw data (colored spline lines), the fit (black lines) according to Eq. (7) and (8) and exponential fits (orange short dashed lines) for bare lipid vesicles with varying gradient factor G = c_out_/c_in,0_. Equal volumes of vesicle suspension (300mM NaCl, 20mM MOPS, pH 7.5) and hyperosmotic solution (c_Δ,sucrose_: 125mM – red line; 200mM – green line; 300mM – yellow line; 500mM – blue line; 750mM – pink line) were mixed at 4°C.


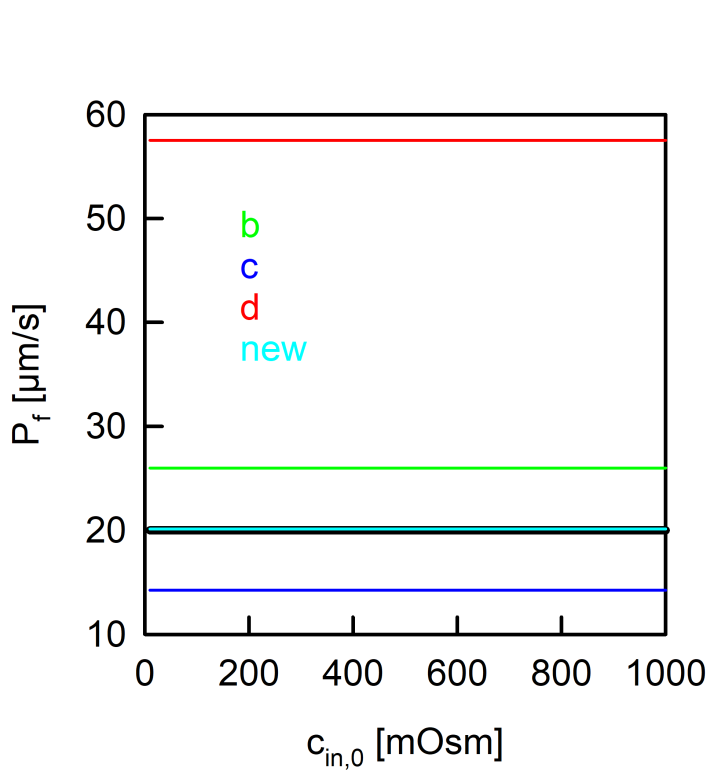


**Supplementary Figure S2. Water permeabilities are independent of the absolute solute concentrations.** P_f_ values calculated from time constants τ of exponential fits to the computed analytical solution (black short dashed line) using different models (Fig. 1, b-new) are shown in color (color coded). c_in,0_ and c_out_ are varied at a fixed gradient factor G = c_out_/c_in,0_ of 1.5.


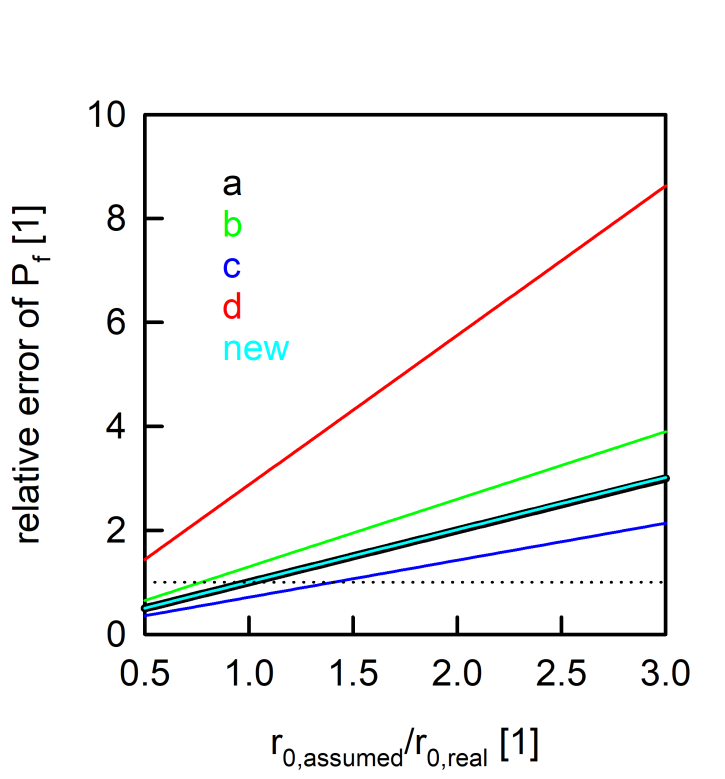


**Supplementary Figure S3. The relative error of permeabilities linearly depends on the relative error of vesicle radius r_0_.** Under- or overestimation of r_0_ during the calculation of P_f_ directly scales with an under- or overestimation of P_f_. The different models are color coded as in the previous figures.
